# A Novel Ziegler–Natta-Type Catalytic System—TiCl_4_/2,2′-Dimethoxy-1,1′-Binaphthalene/Et_3_Al_2_Cl_3_/Bu_2_Mg for Production of Ultrahigh Molecular Weight Polyethylene Nascent Reactor Powders, Suitable for Solvent-Free Processing

**DOI:** 10.3390/polym10111281

**Published:** 2018-11-17

**Authors:** Olga A. Serenko, Mikhail I. Buzin, Vladislav A. Tuskaev, Svetlana C. Gagieva, Nikolay A. Kolosov, Dmitrii A. Kurmaev, Tatyana F. Savel’eva, Evgenii K. Golubev, Sergey V. Zubkevich, Viktor G. Vasil’ev, Galina G. Nikiforova, Alexander A. Korlyukov, Boris M. Bulychev

**Affiliations:** 1 A. N. Nesmeyanov Institute of Organoelement Compounds, Russian Academy of Sciences, 28 ul. Vavilova, 119991 Moscow, Russia; oserenko@yandex.ru (Q.A.S.); buzin@ineos.ac.ru (M.I.B.); sgagieva@yandex.ru (S.C.G.); vim@ineos.ac.ru (T.F.S.); jeckagolubev@gmail.com (E.K.G.); viktor@ineos.ac.ru (V.G.V.); ggn@ineos.ac.ru (G.G.N.); alex@xrlab.ineos.ac.ru (A.A.K.); 2 Department of Chemistry, M. V. Lomonosov Moscow State University, 1 Leninskie Gory, 119992 Moscow, Russia; kolosovna@mail.ru (N.A.K.); dmitrykurmaev@mail.ru (D.A.K.); zubkevichsergey@gmail.com (S.V.Z.); b.bulychev@highp.chem.msu.ru (B.M.B.); 3 Enikolopov Institute of Synthetic Polymer Materials, Russian Academy of Sciences, Profsoyuznaya Str., 70; 117393 Moscow, Russia

**Keywords:** Ziegler-Natta catalysts, UHMWPE, solvent-free processing

## Abstract

A series of ultrahigh molecular weight polyethylenes with viscosity-average molecular weights in the range of 1.6–5.6 × 10^6^ have been prepared by using a novel Ziegler–Natta-type catalytic system—TiCl_4_/2,2′-dimethoxy-1,1′-binaphthalene/Et_3_Al_2_Cl_3_/Bu_2_Mg at different temperatures (*T*_poly_) in the range between 10 and 70 °C in toluene. The morphology of the nascent reactor powders has been studied by scanning electron microscopy, wide-angle X-ray diffraction, and the DSC melting behavior. Polymers are suitable for the modern processing methods—the solvent-free solid-state formation of super high-strength (tensile strength over 1.8–2.5 GPa) and high-modulus (elastic modulus up to 136 GPa) oriented film tapes. With decrease of *T*_poly_, the drawability of the reactor powders increased significantly.

## 1. Introduction

Overall, most of industrial processes for the preparation of polyolefins are carried out using heterogeneous Ziegler–Natta (ZN) catalytic systems [1,2]. One of the important products of these syntheses is ultrahigh molecular weight polyethylene (UHMWPE), that represents one of the most prospective constructional materials, because it exhibits a unique complex of physical and mechanical properties: low coefficient of friction, low moisture adsorption, high chemical resistance, no known toxicity effects, high impact elasticity, at record-breaking low brittle temperatures (down to −200 °C), which allows making ware based on this material for the operation under extreme conditions. One of the most important advantages of this polymer is the possibility of its processing into high-strength and high-modulus films or film tapes. Besides, UHMWPE belongs to the most widely available and cheapest of polymeric materials. [3]. However, there is a downside: high molecular weight and a large number of entanglements between chains are responsible for the extremely high viscosity of the polymer melt, which requires the use of special methods for processing of the reactor powder into high-strength fibers.

The main method for manufacturing high-strength and high-modulus UHMWPE fibers (strength above 2.5 GPa, modulus of elasticity up to 150 GPa) is implemented by gel-spinning with subsequent orientation stretching. The main disadvantage of this method is the need for large quantities of high-boiling solvents, and the associated high costs for its regeneration [3]. The developed method of solid-phase processing of UHMWPE reactor powders in high-strength films and tapes [4,5,6] certainly has a number of serious technological and economic advantages in comparison with gel-spinning. However, this method of processing imposes rather strict requirements for the structure, morphology, and dispersion of the UHMWPE nascent reactor powders (NRP). Single-site non-metallocene catalysts have recently been used to produce reactor powders of the required quality [7,8,9,10,11,12,13], however, in our opinion, the potential of simpler Ziegler–Natta systems is far from exhausted.

Modern ZN systems are multicomponent mixtures that contain a transition metal compound (mainly group 4), non-transition organometallic compounds (activators), specially prepared anhydrous magnesium chloride or other solid supports, and external and internal donors (organic or organoelement compounds, for example, diesters, diethers, and silanes). The distinct mechanism of catalysis, the compositions of active sites, oxidation states of transition metals, the role of magnesium chloride, and donors in the formation of catalytic active sites, have not been unequivocally established and, to this day, remain the subjects of discussion [14]. It is quite obvious that all these problems cannot be solved comprehensively, even with the use of the most modern research methods. Basically, for solving these questions, the methods of quantum chemistry are used. It should be noted, however, that the introduction of internal donors into the catalytic system transforms the “classical” version of ZN system into a latent variant of post-metallocene catalyst. In other words, internal donors should be considered as ligands, forming complexes with transition metal compounds that further form active catalytic sites and which substantially determine not only the isospecificity of the polymerization reaction but, also, the activity of catalytic systems, the molecular weight, the molecular weight distribution, and the microstructure of the obtained polymers [15].

The replacement of “classical” donors—phthalates—with 1,3-diethers, led to the development of the 5th generation of ZN-catalysts, with extremely high activity and regioselectivity control, without the need for any external donor [16]. In the first approximation, systems based on TiCl_4_ complexes with simple cyclic ethers, for example, TiCl_4_(THF)_2_ and TiCl_3_(THF)_3_, can be classified as post-metallocene catalysts. These complexes, in the presence of organomagnesium and organoaluminum compounds, are moderately active in the polymerization of ethylene, and lead to the formation of UHMWPE powders which can be processed by the solid-phase method [17,18,19]. However, the organic ligands in these complexes can occupy any of the six sites in the octahedron and, thereby, produce a certain set of active sites upon activation. In this respect, chelating ligands, such as diethers, have less freedom in coordination patterns and form sterically rigid structures.

Research aimed at developing of new internal donors among aliphatic ethers is in progress [20,21,22], but the use of alkoxyarenes in this role has been studied to a lesser extent. It is noted that the use of anisole instead of the “classical” diethyl phthalate leads to an increase in the activity of ZN catalysts in the copolymerization of ethylene and hexene-1 [23] and, also, the polymerization of hexene-1 [24]. Since the internal donors with two oxygen atoms have been found to be more effective than single oxygen-containing donors [25], in the present work, we used 2,2′-dimethoxy-1,1′-binaphthalene as a neutral bidentate chelating ligand, capable of forming an octahedral titanium complex. In the first approximation, it can be regarded as an analogue of the *bis*-tetrahydrofuran complex, but with the difference that the firmly bound oxygen atoms in the ligand will make the whole structure of the complex more rigid.

The main aim of the present work was to develop an ethylene polymerization technique on the catalytic system TiCl_4_/2,2′-dimethoxy-1,1′-binaphthalene/Et_3_Al_2_Cl_3_/Bu_2_Mg, that could produce the UHMWPE reactor powder suitable for processing into high-strength and high-modulus oriented film tapes using a solvent-free (solid-phase) method.

## 2. Materials and Methods

All manipulations with air-sensitive materials were performed with rigorous exclusion of oxygen and moisture in oven-dried Schlenk glassware on a dual manifold Schlenk line, interfaced to a high-vacuum line. Argon and ethylene of special-purity grade (Linde gas) were dried by purging through Super Clean™ Gas Filters. Toluene and pentane were distilled over Na/benzophenone. The water contents in these solvents were periodically controlled by Karl Fischer coulometry using a Methrom 756 KF apparatus. CDCl_3_ was stored over 4 Å sieves. Unless otherwise noted, all reagents were purchased from Sigma-Aldrich (Milwaukee, WI, USA). 2,2′-Dimethoxy-1,1′-binaphthyl was prepared by using the following literature procedures [26], and its ^1^H and ^13^C NMR spectra were found to match the published data.

### 2.1. Synthesis of Pre-Catalyst

**[TiCl_4_(L)], where L is 2,2′-dimethoxy-1,1′-binaphthalene**. A Schlenk tube fitted with a magnetic stirring bar was charged under argon atmosphere with ligand **2** (157.0 mg, 0.5 mmol), toluene (10 mL), and TiCl_4_ (0.05 mL, 0.5 mmol). The reaction mixture was stirred at room temperature for 8 h. Then, the organic solvent was evaporated; the obtained red powder was washed with pentane and recrystallized from toluene. Yield: mg (63%). anal. calcd for C_22_H_18_Cl_4_O_2_Ti (504) (%): C: 52.44; H: 3.62; Cl: 28.17; O: 6.33; Ti: 9.5%. Found (%): C: 52.81; H: 3,72; Cl: 28.21; Ti: 9.61.

### 2.2. Polymerization of Ethylene

Polymerization of ethylene was carried out in a 400 mL stainless steel reactor (Parr Instrument Co., Moline, IL, USA) equipped with a mechanical stirrer, a temperature controller, and inlets for loading components of catalytic systems and ethylene. The process was conducted at a total ethylene and toluene vapor pressure of 0.7 atm. At first, the reactor was preheated to 100 °C and evacuated for 10 min. Then, toluene (100 mL) and the desired amount of a co-catalyst (mixture of Et_3_Al_2_Cl_3_/Bu_2_Mg) were loaded in the reactor. The reactor was thermostatically controlled at the specified temperature (10–70 °C), and the reaction mixture was saturated with ethylene. Polymerization was initiated by addition of pre-catalyst—[TiCl_4_(L)], where L is 2,2′-dimethoxy-1,1′-binaphthalene—to the reaction mixture. The pressure of ethylene was maintained constant during polymerization. Polymerization was stopped by addition of ethanol (20 mL) and 10% HCl solution in water (10 mL) to the reactor. The polymer was filtered off, washed several times with water–ethanol mixture, and dried under vacuum at 50–60 °C, until a constant weight was achieved.

### 2.3. Polymer Evaluation Methods

Viscosity-average molecular weight of synthesized UHMWPE samples was calculated with the Mark–Houwink equation: *M*_v_ = 5.37 × 10^4^ [*η*]^1.37^ [3], where *M*_v_ = viscosity-average molecular weight (g/mol); [*η*] = intrinsic viscosity in decalin at 135 °C (dl/g); [*η*] = (2*η*_sp_ − 2*lnη*_r_)^1/2^/0.056 (*η*_sp_—specific viscosity decalin at 135 °C; *η*_r_—relative viscosity in decalin at 135 °C); *η*_r_ = *η*_sp_ + 1.

Scanning electron microscopy investigations of morphologies of nascent reactor powders were carried out with a high resolution FEG SEM (Carl Zeiss Leo 1530 VP, Columbus, OH, USA) operated at 5 kV. As-polymerized particles were carefully deposited on SEM stubs, and the samples were coated with gold by a sputtering technique.

DSC was performed by a differential scanning calorimeter DSC-822e (Mettler-Toledo, Columbus, OH, USA) at a heating/cooling rate of ±10 °C/min.

X-ray data for nascent reactor powders were measured in the Center for Molecular Composition Studies of INEOS RAS on the Bruker D8 Advance diffractometer (Ge (111) monochromator, *λ*[CuK_α_1] = 1.5406 Å).

Mechanical characteristics of the oriented materials, prepared using the synthesized polymers, were evaluated by means of obtaining the oriented tapes by a solid-state processing of UHMWPE nascent reactor powders. Monolithic tapes that were uniform over the entire length (100 microns in thickness and 10 mm in width) were formed at a pressure and shear deformation below the polymer melting point (124–126 °C). The tapes were subjected to uniaxial drawing while using Spinline Daca equipment (Santa Barbara, CA, USA). The drawing temperature was set 4 °C below the polymer melting point. The mechanical characteristics of the tapes were measured with a Hounsfield H1KS machine at the gauge length of the tested samples of 120 mm with 2 mm/min initial deformation rate. The reported values are the average of at least 8 samples.

## 3. Results and Discussion

It is well known that the properties of UHMWPE obtained on various catalytic systems depend both on the chemical composition of these systems, and on the conditions of the polymerization process. Thus, using bis(phenoxyimine)titanium dichloride complexes activated by methylaluminoxanes, with an increase in the polymerization temperature from −15 to 20 °C, the molecular weight of UHMWPE increases [27] while, on similar systems, an increase in the process temperature from 20 to 60 °C is accompanied by a significant decrease in the *M*_v_ of the polymers [28]. When using a catalytic system {Bu_2_Mg–TiCl_4_–THF–Et_3_Al} with increasing temperature, from 10 to 60 °C, the molecular weight of the UHMWPE also decreases [17]. On the classical high-activity Ziegler catalyst, no apparent relationship between the molecular weight of polymer and the synthesis temperature (from 20 to 90 °C) was established [29].

Ethylene polymerization experiments using a {TiCl_4_/2,2′-dimethoxy-1,1′-binaphthalene/Et_3_Al_2_Cl_3_/Bu_2_Mg} catalytic system have been carried out in 100 mL of toluene with 5 × 10^−6^ mol of catalyst at a constant ethylene pressure of 0.7 atm for 30 min, [Ti]/[Et_3_Al_2_Cl_3_]/[Bu_2_Mg] = 1:300:100. Catalytic properties of this system in ethylene polymerization depending on temperature are summarized in Table 1.

**Figure 1:**
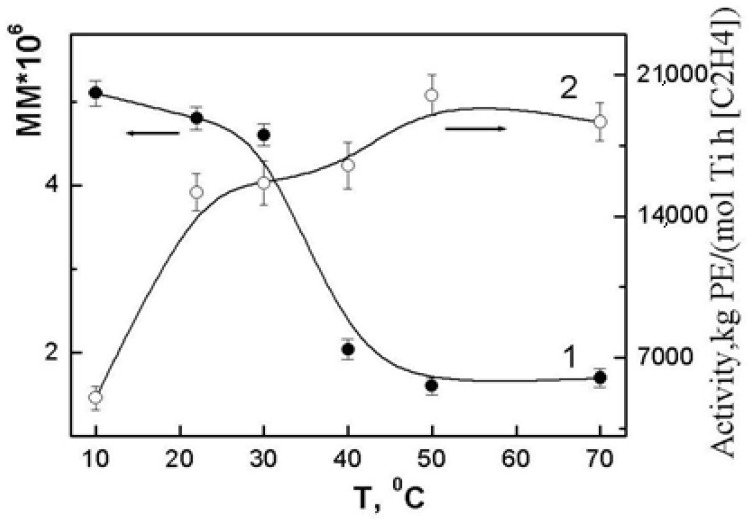
Effect of polymerization temperature on the *M*_v_ polymers (1) and the activity of the catalytic system (2).

Polymerization of ethylene by the {TiCl_4_/2,2′-dimethoxy-1,1′-binaphthalene/Et_2_Al_2_Cl_3_/Bu_2_Mg} catalytic system in the entire operating temperature range leads to polymers with the *M*_v_ characteristic for UHMWPE. Figure 1 shows the dependence of viscosity-average molecular weights on the polymerization temperature, which has the shape of a step. In the range from 10 to 30 °C, the *M*_v_ varies insignificantly, from 5.6 × 10^6^ to 4.6 × 10^6^. A further increase in the synthesis temperature to 40 °C results in a sharp, by half, decrease in the *M*_v_ of the polymer. In the interval from 40 to 70 °C, the change in the *M*_v_ is low. In contrast to the temperature dependence of the molecular weights of the polymers, the activity of the catalytic system increases monotonically with increasing temperature (Figure 1).

**Figure 2:**
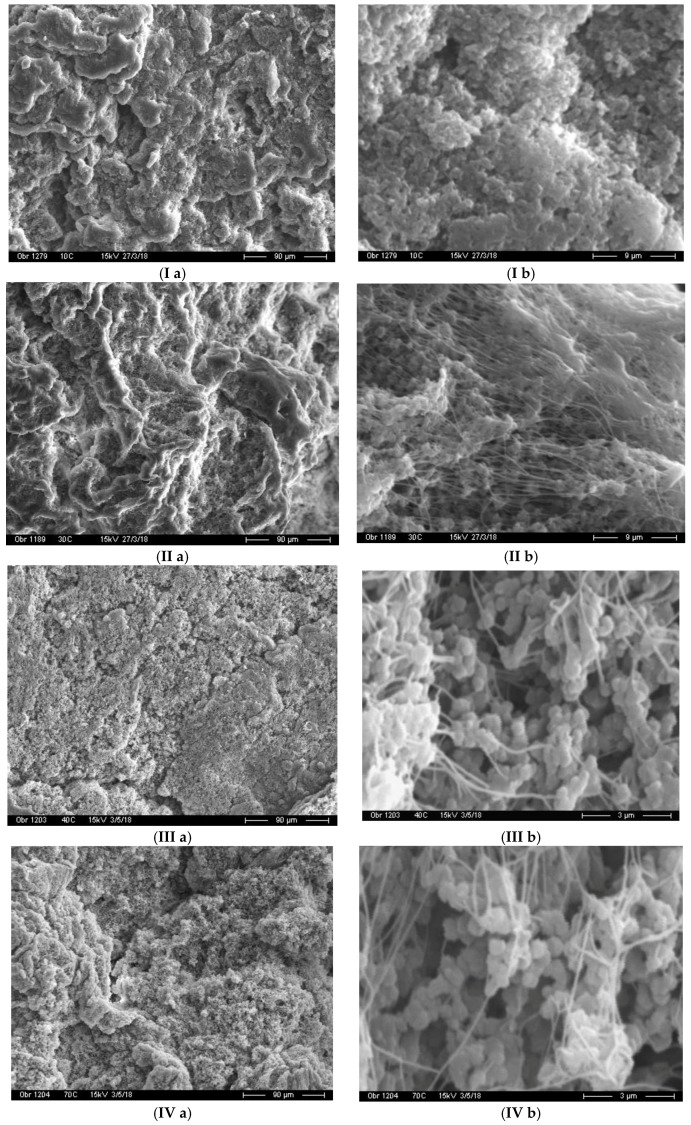
Scanning electron micrographs of a series of nascent reactor powders, prepared at different *T*_poly_: 10 °C (**I a,b**); 30 °C (**II a,b**); 40 °C (**III a,b**); and 70 °C (**IV a,b**).

It is well known that drawability of UHMWPE highly depends on its supramolecular structure. To examine the morphologies of these powders, SEM observations were made (Figure 2). It is apparent that *T*_poly_ affects not only the *M*_v_ of the polymer but, also, the surface morphology of the reactor powders. The polymer synthesized at 10 °C has a sufficiently low-porous, uniform surface consisting of fine, densely packed particles 300–600 nm in size (Figure 2I(a,b)). This type of structure is commonly called broccoli-like [30]. On the surface of powders obtained at 30 °C, both dense and friable areas are observed, in the latter, small particles that are close in shape to spherical, or clusters of particles are linked together by fibrils (Figure 2II(a,b)). A fundamental change in the morphology of UHMWPE powders occurs at the synthesis temperature of 40 °C or higher. In this case, their surface can be described as porous, fluffy. In addition, there are no zones with a dense packing of spherical particles on the surface, while the content of fibrils increases clearly (Figure 2III(a,b)). At *T*_poly_ = 70 °C, the amount of fibrils becomes comparable with the spherical particles, forming a stretched fibrillar grid (or “cobweb” structures), entangling agglomerates of small spherical particles (Figure 2IV(b)). Thus, the synthesis of UHMWPE on the considered catalytic system at elevated temperatures promotes the formation of highly agglomerated powders of spherical and fibrillar forms.

An analogous dependence of the morphology of the UHMWPE reactor powders, obtained on a highly active ZN catalyst, on the synthesis temperature, is described in [29]. It is assumed that the origin of such fibrous structures is an internal tensile stress in the polymer particle that increases during polymerization as its mass increases, under the condition of high activity of the catalyst [31,32,33].

Explicit differences in the properties of reactor powders, obtained at temperature ranges of 10–30 °C and 40–70 °C, are manifested not only in the *M*_v_ values and surface morphology but, also, in thermal characteristics of the polymer (Table 2). The melting points of reactor powders synthesized in the range of temperatures from 10 to 30 °C, and having *M*_v_ values of (4.6–5.6) × 10^6^ Da (the first group) are 141–142 °C, and the degree of crystallinity is 72–75%. The melting temperatures of reactor powders obtained at higher temperatures (40–70 °C) are slightly lower—137–138 °C—and the content of the crystalline phase is 69%.

At the second heating run, the melting temperatures are equalized and reduced to 136–137 °C, their degree of crystallinity also decreases, and the heat of fusion coincides with the heat of crystallization.

**Figure 3:**
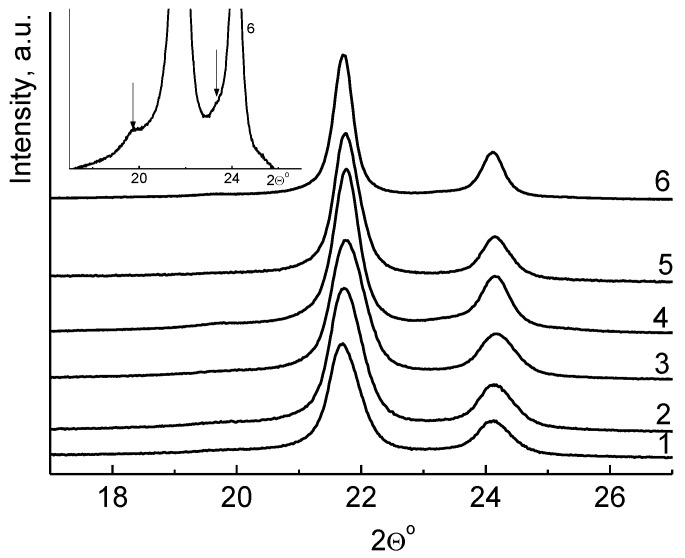
WAXD profiles for a series of ultrahigh molecular weight polyethylene (UHMWPE) reactor powders, obtained at 10 (1), 22 (2), 30 (3), 40 (4), 50 (5), and 70 °C (6), and a typical enlarged fragment of the WAXD profile 6 (b). The arrows indicate reflexes corresponding to the monoclinic phase (010) m and (200) m.

The crystalline forms of these powders have also been analyzed by WAXD (wide angle X-ray diffraction). Figure 3 compares the WAXD patterns for the compacted UHMWPE powders obtained at the temperature range of 10–70 °C. The orthorhombic reflections (peaks (110°) and (200°)) are dominant for all powders, regardless of the polymerization temperature. Figure 3b shows a typical enlarged fragment of the X-ray powder diffraction pattern. It can be seen that there are reflexes characteristic for the monoclinic phase of polyethylene—a diffuse peak at an angle of 19.5° (010) m, and a shoulder at 23.1° (200) m. The monoclinic phase is present in all samples regardless of the temperature of their synthesis, but its content is extremely small, which prevents a correct evaluation of its content in reactor powders.

It has been shown [34] that, with an increase in the drawing ratio of UHMWPE films, their crystallinity increases, including the content of the monoclinic phase, which ultimately leads to a significant improvement in mechanical characteristics. However, since the monoclinic crystals easily transform into the normal orthorhombic crystals at heating to 100 °C [35,36,37], there is no reason to believe that its presence in the reactor powder will have a significant impact on the orientational drawing process of the polymer.

Thus, the considered catalytic system, selected concentrations of pre-catalyst and activators, Ti/Al/Mg ratio, as well as polymerization conditions (temperature and pressure), make it possible to obtain reactor powders whose morphology and molecular weights determine the possibility of processing into orientated film tapes according to the method proposed in [8]. Indeed, we have obtained film tapes by direct solvent-free molding of reactor powders at an elevated temperature below polymer melting point, with subsequent uniaxial stretching, which can be attributed to typical high-strength high-modulus polymeric materials, by their characteristics. Table 3 shows the degree of drawing of oriented film tapes and their mechanical characteristics. The highest drawing ratio has been achieved during the processing of reactor powders obtained at 10, 22, and 30 °C. In this case, the maximum value of the modulus of elasticity was 136 GPa, and the maximum strength value was 2.5 GPa. The draw ratios for films from reactor powders synthesized at 40 and 70 °C does not exceed 20; values of modulus of elasticity and strength are minimal. 

Obviously, low values of the drawing degree of film tapes from these reactor powders are a consequence not only to smaller values of the polymers *M*_v_, but also to their morphology. The presence of fibrillated elements in the UHMWPE reactor powders clearly prevents uniform distribution of stress in the sample during the orientation drawing and, as a result, the production of film tapes with increased strength characteristics.

Table 4 shows the results of calorimetric studies of orientated films, which generally follow the patterns noted in the study of reactor powders. 

**Figure 4:**
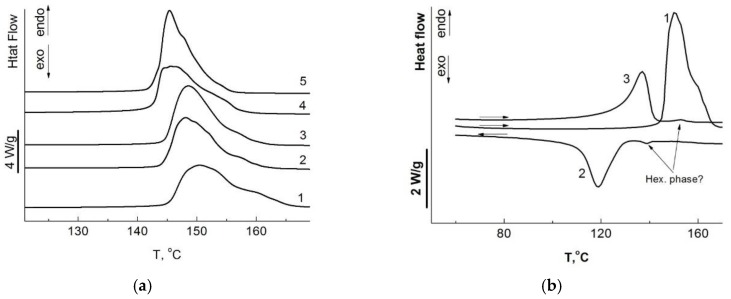
(**a**) DSC traces for UHMWPE films obtained from reactor powders synthesized at 10 (1), 22 (2), 30 (3), 40 (4), and 70 °C (5) on first heating at a heating rate 10 °C/min in argon; (**b**) DSC traces for UHMWPE films obtained from reactor powders synthesized at 10 °C on first heating (1), cooling (2), and reheating (3) at a heating/cooling rate ±10 °C/min in argon.

The main melting peak of oriented filaments at the first heating is located in the temperature range of 140–150 °C (Figure 4a, Table 4), which is significantly higher than for the initial reactor powders. The position of the main melting peak during the first heating decreases with increasing synthesis temperature. The form of the endothermic effect of melting UHMWPE samples obtained in this work is noteworthy (Figure 4). Their asymmetry, most clearly manifested in the thermogram during the first heating run of the film obtained from UHMWPE powder synthesized at 10 °C, indicates the coexistence of two phases. Most likely, the metastable hexagonal phase is the high-temperature phase, the formation of which was previously recorded in UHMWPE fibers obtained in the process of gel-forming and orientational stretching [38], or in UHMWPE samples subjected to high pressure [39].

A similar endothermic peak was observed earlier on DSC thermograms at the second heating of UHMWPE fibers [40]. According to the literature, this temperature corresponds to the melting of the hexagonal crystalline phase. 

## 4. Conclusions

Based on the results of a systematic study of the influence of polymerization temperature on the properties of UHMWPE reactor powders, in particular, on the possibility of their solid-phase processing, the following conclusions can be drawn. 

The use of a Ziegler–Natta catalytic system {TiCl_4_/2,2′-dimethoxy-1,1′-binaphthalene/Et_3_Al_2_Cl_3_/Bu_2_Mg} provides high yields of UHMWPE, with the possibility of regulating its molecular weight and morphology of the powder surface. 

An internal donor—2,2′-dimethoxy-1,1′-binaphthalene—is able to form a stable chelate with titanium tetrachloride, wherein its structure is univariant. This situation is radically different from the catalytic systems with monodentate ligands containing ethereal oxygen donors, for example, THF, where these molecules can occupy different positions in the octahedral complex of TiCl_4_(thf)_2_. A consequence of this diversity can be the multicenter nature of catalysts and the reduction of all parameters of the catalytic process, including the quality of the resulting polymer. Unfortunately, we have not been able to determine MMD to confirm this assumption but, from the results obtained in this work, it follows that the activity of the system with a diether ligand, ceteris paribus, more than twice exceeds the activity of the system based on TiCl_4_(thf)_2_. The mechanical characteristics of the oriented UHMWPE film tapes obtained in this paper also exceed those obtained with the {TiCl_4_–THF–Bu_2_Mg–Et_3_Al_2_Cl_3_} system [19], although the melting points, the degrees of crystallinity, and the molecular masses remain close to each other in both cases.

The high activity of this catalytic system assumes the synthesis of the polymer at temperatures not higher than 30 °C. The morphology of the obtained reactor powders makes it possible to use the method of solvent-free processing to obtain high values of the mechanical properties of the oriented materials. 

An increase in the synthesis temperature above 30 °C negatively affects both the *M*_v_ and the morphology of the resulting powder. The fibrillar structure of the UHMWPE powders formed at increased polymerization temperature prevents the achievement of high degrees of orientational drawing, which, as a result, does not allow achieving the desired high-strength characteristics of the films.

## Figures and Tables

**Table 1:** Ethylene polymerization by {TiCl_4_/2,2′-dimethoxy-1,1′-binaphthalene/Et_3_Al_2_Cl_3_/Bu_2_Mg} catalytic systems ^a^.

Entry	*T*_poly_, °C	Activity, kg polyethylene /(mol Ti h atm)	Activity, kg polyethylene /(mol Ti h [C_2_H_4_])	*M*_v_, ^b^ 10^6^ Da
1	10	1200	4970	5.6
2	22	3085	15,200	4.8
3	30	3097	15,260	4.6
4	40	2630	16,283	2.0
5	50	2485	20,470	1.6
6	70	2000	18,700	1.7

^a^ Polymerizations have been carried out in 100 mL of toluene at a constant ethylene pressure of 0.7 atm for 30 min, [Ti] = 5 × 10^−6^ mol; [Ti]/[Et_3_Al_2_Cl_3_]/[Bu_2_Mg] = 1:300:100. ^b^ Viscosity-average molecular weights were calculated with the Mark–Houwink equation: *M*_v_ = 5.37·10^4^ [*η*]^1.37^.

**Table 2:** DSC data and crystallinity of nascent reactor powders.

*T*_poly_, °C	First Heating			Cooling			Reheating		
	*T*_m1_, °C	Δ*H*_m1_, J/g	*α*_m1_, %	*T*_c_, °C	Δ*H*_c_, J/g	*α*_c_, %	*T*_m2_, °C	Δ*H*_m2_, J/g	*α*_m2_, %
10	142	224	75	118	123	41	136	120	40
22	141	216	72	116	130	43	136	130	44
30	142	220	74	115	131	44	137	132	44
40	138	207	69	117	149	50	137	149	50
50	138	206	69	116	156	52	136	154	52
70	137	207	69	116	165	55	137	165	55

**Table 3:** Mechanical properties of UHMWPE oriented film tapes.

*T*_poly_, °C	*M*_w_, 10^6^ Da	Drawing Ratio	*E*, GPa	*σ*, GPa	*ε*, %
10	5.6	32	136	2.3	2.0
22	4.8	32	136	2.4	2.2
30	4.6	28	125	2.5	2.5
40	2.0	20	100	1.8	2.5
70	1.7	20	96	1.8	3.3

**Table 4:** DSC data of oriented film tapes.

*T*_poly_, °C	First Heating		Cooling		Reheating	
	*T*_m1_, °C	Δ*H*_m1_, J/g	*T*_c_, °C	Δ*H*_c_, J/g	*T*_m2_, °C	Δ*H*_m2_, J/g
10 (32) *	150	267	119	119	136	124
			139 **	0.05 **	153	2,0
22 (32)	148	265	117	140	136	142
30 (28)	148	271	119	129	136	131
40 (20)	145	259	-	-	136	155
70 (20)	145	253	114	156	137	164

* The values of the draw ratio of film samples are given in brackets. ** The denominator shows the thermophysical characteristics of the peak, presumably related to the hexagonal phase.
